# Supplementation of *Hermetia illucens* Larvae in Poultry By-Product Meal-Based Barramundi, *Lates calcarifer* Diets Improves Adipocyte Cell Size, Skin Barrier Functions, and Immune Responses

**DOI:** 10.3389/fnut.2020.613158

**Published:** 2021-01-14

**Authors:** Md Reaz Chaklader, Janet Howieson, Ravi Fotedar, Muhammad A. B. Siddik

**Affiliations:** ^1^School of Molecular and Life Sciences, Curtin University, Bentley, WA, Australia; ^2^Department of Fisheries Biology & Genetics, Faculty of Fisheries, Patuakhali Science and Technology University, Patuakhali, Bangladesh

**Keywords:** *Vibrio harveyi*, insect larvae, adipocyte cell, neutral mucins, immune function, barramundi

## Abstract

A 6-week feeding trial was performed to examine the effects of supplementing *Hermetia illucens* (HI) larvae meal when fishmeal (FM) was replaced with poultry by-product meal (PBM) in juvenile barramundi, *Lates calcarifer* diet. The effect was evaluated in terms of barramundi growth, filet quality, internal tissue structure, serum biochemistry, skin neutral mucins, immune response, and resistance to *Vibrio harveyi*. Three isonitrogenous (48% crude protein) and isolipidic (18% crude lipid) diets: an FM-based diet (control) and two diets containing 60 and 75% of PBM supplemented with 10% HI larvae (60PBM + HI and 75PBM + HI) were formulated. A total of 225 barramundi, with an average weight of 15.87 ± 0.14 g, were randomly distributed into nine tanks, each holding 25 fish. There were no significant effects of test diets on growth, but feeding HI-supplemented PBM diets significantly increased the survival rate. A significantly reduced intraperitoneal fat index in HI-supplemented-PBM-fed fish was correlated to a decreased size of peritoneal adipocytes. The observation of no histopathological alteration of the liver in the HI-supplemented-PBM-fed fish was further supported by significant alterations in serum biochemistry, in particular, a decreasing tendency of alanine transaminase, glutamate dehydrogenase, and total bilirubin. A 14-day challenge with *V. harveyi* indicated that HI-supplemented PBM diets reduced the infection rate in barramundi. After 24 h of infection, increased serum (lysozyme) and skin barrier functions, down-regulation of interleukin-1beta, and upregulation of interleukin-10 were found in HI-supplemented-PBM-fed fish.

## Introduction

Insects are the prey of many carnivorous and omnivorous fish in their natural environment (1) and hence could be a nutritionally suitable and potential source of sustainable alternative animal protein ingredients for aquacultured species. Insects are characterized by high quantity (60–80%) and quality of protein, ability to grow on waste and by-products, and fast growth with a lower risk of transmission of zoonotic diseases and may contain native bioactive peptides with anti-microbial, anti-fungal, and anti-viral functions (1–6). In particular, insect larvae belonging to the Diptera family contain essential amino acid compositions that are similar to fishmeal (FM) (1).

*Hermetia illucens* (HI) larvae of the Diptera family, commonly known as black soldier fly, have begun to play an important role in aquafeed production. HI larvae can dwell in harsh environments, infested with bacteria and fungi, leading to the production of low molecular weight antimicrobial factors, which have antifungal and antibacterial effects against Gram-positive and Gram-negative bacteria (3, 7). HI larvae are also characterized by the presence of chitin and high levels of medium-chain fatty acids, especially lauric acid (C12:0) (8). Chitin and chitin derivatives are reported to have an immunomodulating capacity in fish (9), and lauric acid is well-documented for its antibacterial and antiviral activity (10). Therefore, complete HI larvae as a supplement could provide a crucial value-added role in the diet of aquaculture species, especially for carnivorous fish.

Over the last decades, significant efforts have been made by commercial feed producers and fish farmers to investigate the use of animal by-product meal, in particular, poultry by-product meal (PBM), to replace fishmeal (FM), an expensive and often considered unsustainable source of protein in the diet of carnivorous fish (11–14). PBM, an economical and arguably a more sustainable aquafeed ingredient, contains high-quality protein (58–65%) and amino acids similar to FM and is also a good source of vitamins and minerals (15–17). A number of studies regarding the inclusion of PBM in aquadiets have been published over the last decade, though the findings are still controversial in terms of inclusion levels of PBM and have tended to investigate the replacement of FM protein with PBM protein without any supplementation. Some studies were able to substitute up to 100% FM protein with PBM in humpback grouper (18), Nile tilapia, *Oreochromis niloticus* (19, 20), and hybrid striped bass, *Morone chrysops* × *Morone saxatilis* (21) without deleterious effects on growth. In contrast, inclusion levels of 50% and greater were reported to impose adverse effects on many fish species including tench, *Tinca* (22), black sea turbot, *Psetta maeoticus* (23), and cobia, *Rachycentron canadum* (24). In addition, replacing FM with PBM negatively influenced the immune response of largemouth bass, *Micropterus salmoides* (25, 26).

*Lates calcarifer*, commonly known as barramundi, is one of the highly valued euryhaline carnivorous fish species used for commercial farming both in marine and freshwater environments, particularly in Australia and some other Southeast Asian countries including Indonesia, Philippines, Malaysia, Thailand, and Taiwan (27). Intensive farming of barramundi in marine net cages and in freshwater ponds has accelerated disease outbreaks in Southeast Asia (28). Vibriosis caused by *V. harveyi* is one of the major problems in barramundi farming, causing a major loss through mortalities (28, 29). Much research has been conducted to develop alternative therapeutics for barramundi, including the use of plant herbs and fish protein hydrolysate (14, 28, 30, 31). However, disease resistance against bacterial pathogens in the aquatic system in response to insect larvae supplementation with animal by-products remains unknown.

The mucosal surfaces of fish, such as gill, skin, and gastrointestinal tracts, are important sites for microbial exposure, forming a thin biochemical and physical barrier between the external environment and the internal milieu to protect fish from any pathogenic invasion (32). Among all mucosa-associated lymphoid tissue, skin-associated mucosa, serving as an anatomical and physiological barrier against pathogenic microorganisms encountered, plays a crucial role in the immune system of fish (32). Recently, research on gut-associated lymphoid tissue has been intensified in relation to dietary parameters (33, 34); however, fish skin mucosal immunity is a new interest and rarely studied in finfish aquaculture (32). Therefore, it is of interest to study how dietary alteration and challenge with pathogens may induce skin-relevant immunity in fish.

To date, insect supplementation with animal by-products in the diets of carnivorous fish has not been intensively assessed. Hence, the present work was aimed to evaluate the effect of incorporating PBM as a replacement of FM, along with supplementation of HI larvae meal, on the biological and physiological response of barramundi through a multidisciplinary approach integrating biometric, histological, biochemical, molecular, immunological, and bacterial challenge analyses.

## Materials and Methods

### Ethics Statement

All experimental procedures involving fish were performed in a recirculating aquaculture system at Curtin Aquatic Research Laboratory (CARL) in Curtin University, Australia, in strict accordance with the Australian Code of Practice for the care and use of animals for scientific purposes, and following review and approval by the Curtin University Animal Ethics Committee (approval number: ARE2018-37). Experimental procedures were dedicated to minimizing stress, pain, and discomfort to the fish, including using an anesthetic (AQUI-S®, 8 mg/L) and, for euthanasia, an overdose of AQUI-S® (175 mg/L). Such practices were outlined in the protocol of the CARL standard operating procedures (SOP) of anesthetizing and euthanizing of fish.

### Diets

Three isonitrogenous (48% crude protein) and isolipidic (18% crude lipid) diets were formulated based on locally available ingredients to meet the nutritional requirement of barramundi (Table 1). All the ingredients were procured from Specialty Feeds, Glen Forrest Stockfeeders, 3150 Great Eastern Highway, Glen Forrest, Western Australia 6071, with the exception of HI larvae and PBM. Six-day-old HI larvae were received from Future Green Solution having been cultivated in plastic trays and filled with a mixture of carp mince and grain waste (70:30) for 6 days. HI larvae were collected, dried in an oven for 48 h at 60°C, and ground into fine powder by a digital blender (Anko, XJ-12412, China). PBM was kindly provided by Derby Industries Pty Ltd T/A, Talloman Lot Lakes Rd, Hazelmere WA 6055. All the dried ingredients were weighed and mixed homogenously in a food mixer (Hobart Food equipment, Australia), and then oil and distilled water were added to make a dough. Furthermore, 3-mm-long pellets were produced by passing the dough through a mincer; the pellets were then dried in an oven for 36 h at 60°C, broken up by hand after cooling at room temperature, and sealed in plastic bags before storing in the refrigerator until use in the feeding trial. The fatty acid profile of the HI larvae, PBM, and experimental diets are presented in Table 2.

**Table 1:** Feed formulation and nutritional composition of test diets containing two different levels of PBM supplemented with full-fat HI larvae.

	**Control**	**60PBM + HI**	**75PBM + HI**
**Ingredient (g/100 g)**			
FM^a†^	72.00	22.50	11.00
PBM^b‡^	0.00	42.00	52.50
HI larvae^§^	0.00	12.00	12.00
Cod liver oil^a^	0.50	2.50	2.50
Canola oil^a^	1.00	3.00	3.00
Wheat^a^	16.90	8.40	9.40
Wheat starch^a^	7.00	7.00	7.00
Vitamin C^a^	0.05	0.05	0.05
Vitamin premix^a^	0.50	0.50	0.50
Dicalcium phosphate^a^	0.05	0.05	0.05
Lecithin–soy (70%)^a^	1.00	1.00	1.00
Salt (NaCl)	1.00	1.00	1.00
**Nutritional compositions**^**c**^ **(%)**			
Dry matter	90.21	91.10	90.15
Crude protein	47.88	47.93	47.93
Crude lipid	17.59	17.69	17.77
Ash	13.25	11.85	10.98

a *Purchased from Specialty Feeds, Glen Forrest Stockfeeders, 3150 Great Eastern Highway, Glen Forrest, Western Australia 6071*.

b *Kindly provided by Derby Industries Pty Ltd T/A, Talloman Lot Lakes Rd, Hazelmere WA 6055*.

c *Determined according to the Association of Official Analytical Chemists (35)*.

† *FM, fishmeal (64.0% crude protein, 10.76% crude lipid, and 19.12% ash)*.

‡ *PBM, poultry by-product meal (67.13% crude protein, 13.52% crude lipid, and 13.34% ash)*.

§ *HI, Hermetia illucens larvae (40.43% crude protein and 17.23 % crude lipid)*.

**Table 2:** Fatty acid (mg/100 g on dry matter basis) composition of the three formulated experimental diets and HI larvae and PBM.

	**Control**	**60PBM + HI**	**75PBM + HI**	**HI larvae**	**PBM**
C8:0	1.39	1.27	1.19	4.01	0.90
C10:0	0.59	59.86	65.25	539.10	4.10
C11:0	0.00	0.60	0.48	2.00	0.33
C12:0	2.73	990.02	1,143.19	10,058.58	9.39
C13:0	1.53	1.95	1.49	3.03	0.54
C14:0	131.63	367.55	355.86	1,588.87	73.69
C14:1n5	1.52	11.20	11.78	19.27	16.26
C15:0	41.42	32.58	25.44	34.35	15.14
C15:1	1.19	7.04	4.29	0.92	1.92
C16:0	1,161.21	2,019.55	2,085.29	2,596.67	2,336.27
C16:1n7	165.22	437.70	413.84	361.93	540.27
C17:0	118.84	88.15	70.13	124.60	48.17
C17:1	31.53	39.92	27.38	31.52	19.73
C18:0	448.54	596.51	605.39	819.58	762.41
C18:1cis+trans	1,158.94	3,812.07	4,097.72	3,750.13	4,410.64
C18:2 trans	6.95	10.19	10.78	18.92	6.52
C18:2 cis	624.01	1,876.79	2,067.26	2,856.81	1,736.53
C18:3n6	8.84	13.78	18.57	18.31	23.07
C18:3n3	120.20	400.99	420.75	385.84	260.39
C18:4n3	30.30	71.28	64.87	5.63	13.21
C20:0	22.44	30.48	30.53	21.99	25.98
C20:1	79.86	228.40	202.35	21.16	60.84
C20:2	14.57	19.62	18.46	4.02	19.64
C21:0	9.09	12.58	12.40	0.00	10.61
C20:3n6	15.50	35.96	38.95	4.45	56.76
C20:4n6	112.83	132.99	127.41	12.00	180.13
C20:3n3	8.29	8.94	7.12	1.63	4.43
C22:0	14.10	15.15	14.79	5.76	10.01
C20:5n3	178.50	193.65	134.95	11.30	16.79
C22:1n9	9.44	22.90	19.72	1.38	5.73
C22:2	1.05	1.08	0.90	0.00	0.59
C23:0	27.89	37.60	37.47	58.53	47.20
C22:4n6	91.14	35.44	22.67	0.00	4.78
C24:0	0.00	0.00	0.00	0.00	0.00
C22:5n3	63.30	60.47	50.83	2.02	36.67
C24:1	34.51	31.02	24.94	0.00	2.56
C22:6n3	908.53	473.15	310.50	2.60	27.47
∑SFA	1,981.40	4,253.84	4,448.89	15,857.06	3,344.73
∑MUFA	1,482.21	4,590.25	4,802.02	4,186.31	5,057.95
∑PUFA	2,184.01	3,334.31	3,294.01	3,323.54	2,386.97
∑n-3	1,309.12	1,208.46	989.03	409.03	358.96
∑n-6	228.31	218.17	207.59	34.76	264.74
∑n-3/∑n-6	5.73	5.54	4.76	11.77	1.36

*HI, Hermetia illucens; PBM, poultry by-product meal; SFA, saturated fatty acids; MUFA, monounsaturated fatty acids; PUFA, polyunsaturated fatty acids*.

### Fish Husbandry and Management

Four hundred barramundi, averaging 7.25 g, were collected from the Australian Center for Applied Aquaculture Research, Fremantle, Australia, and transported to CARL in an oxygenated esky. Prior to the start of the trial, the fish were housed in two 300-L seawater tanks and fed a commercial diet for 2 weeks twice a day to adapt them to the CARL experimental conditions. Thereafter, 225 barramundis, fasted for 24 h, were divided equally into nine tanks (25 fish /tank), each filled with 250 L of seawater. Each diet was fed by hand until apparent satiation twice a day at 8:00 am and 6:00 pm for 42 days, with each treatment repeated in triplicate. The rearing conditions and facilities during experimentation were maintained as described in earlier studies at CARL (14, 31). Excess diet from each tank was siphoned off 1 h after feeding, oven-dried for 36 h at 60°C, and then weighed to calculate feed intake. Feed consumption was recorded daily, and dead fish, if any, were weighed and recorded. At the end of the trial, all fish were starved for 24 h and thereafter anesthetized with 8 mg/L AQUI-S® before determining the number of fish and the total fish biomass in each tank to calculate survival rate and growth performance. Length and weight data, viscera, and liver were collected from five randomly chosen fish from each tank to determine the biometry indices, such as condition factor and viscerosomatic and hepatosomatic indices.

### Fatty Acid Profile

Fish muscles from four biological replicates and three technical replicates were used for fatty acid analysis. The fish were fileted to produce muscle, which was wrapped with aluminum foil and freeze-dried for 3 days at −48.4°C and 1.9 × 10^−1^ mB. The fatty acid profiling of experimental diets and fish muscle was carried out following the protocol of O'Fallon et al. (36) and Siddik et al. (13). Approximately 0.5 g of the sample was hydrolyzed at 55°C for 1.5 h with 0.1 ml of internal standard (1.2 g non-adecanoic acid in 100 ml chloroform), 0.7 ml of 10 N KOH, and 5.3 ml of methanol. The sample was then methylated at 55°C for 1.5 h with 0.6 ml of 24 N sulphuric acid. The fatty acid was extracted into 1 ml of hexane and then quantified using gas chromatography with flame ionization detection. The column used was a capillary column HP INNOWax GC column (60 m × 0.25 mm ID; film, 0.50 μm), with hydrogen as the carrier gas. Each sample was analyzed in triplicate, and the results were expressed as an average.

### Challenge Test With *V. harveyi*

The challenge trial was conducted according to the protocol of our earlier study (37). Briefly, at the end of the 6-week feeding trial and after collecting all samples, 10 fish from each dietary treatment were retuned back to their respective tanks and injected intraperitoneally by 1-ml syringe fitted with a 27-gauge needle with 0.1 ml of pathogenic *V. harveyi* suspension (LD_50_ = 1.1 × 10^8^ cfu/ml), which was supplied by Diagnostic and Laboratory Services, Department of Primary Industries and Regional Development (DPIRD), 3 Baron-Hay Court, South Perth WA 6151. *V. harveyi* was grown in trypticase soy broth (Oxoid, Basingstoke, UK) for 24 h at 24°C, and the culture was centrifuged (5,000 × g, 15 min) at 4°C before suspending the pellets in phosphate-buffered saline (PBS, pH 7.2) for the challenge trial. Clinical signs of vibriosis in terms of a thick layer of mucus on the body surface, congestion of the fins, and hemorrhages and ulceration of the skin and muscle tissue were observed three times a day (7:00 am, 2:00 pm, and 9:00 pm) for 14 days, and fish with such symptoms of vibriosis were subjected to euthanasia with AQUI-S at 175 mg/L for 20 min according to the protocol of the CARL SOP for euthanizing of fish.

### Histological Analysis

Liver, intraperitoneal fat, heart, and muscle from six euthanized (AQUI-S®, 175 mg l^−1^) fish/treatment (two/replicate) at the end of the trial and also a similar set of skin samples 24-h post-challenge were collected and immediately preserved in 10% neutral buffered formalin. Following preservation, the fragments of all tissue sections were subsequently dehydrated with a series of ethanol washes, cleared by xylene, and embedded in paraffin wax. The tissue wax was then cut into 5-μm slices by microtome, stained with periodic acid–Schiff as per standard histological procedures, and photographed under a light imaging microscope (BX40F4, Olympus, Tokyo, Japan). The average of epidermis (Ep) thickness was measured from three regions of each section, and the number of epidermis goblet cells were counted in 1-mm length of the epidermis, following the methods of Heidarieh et al. (38) and Sheikhzadeh et al. (39).

### Serum Biochemical Analysis

At the end of the feeding and challenge trial, six randomly chosen fish from each treatment (two/replicate) were anesthetized (AQUI-S®, 8 mg l^−1^), and blood was withdrawn from the caudal vein using 1-ml non-heparinized syringes fitted with 22-G needles and then kept at room temperature for 4 h until coagulation. Serum was obtained from coagulated blood by centrifugation at 3,000 rpm × 15 min at 4°C and immediately stored at −80°C prior to the analysis of serum biochemical parameters and immune parameters.

The serum biochemical assays for two biological replicates and three technical replicates were performed as described in our previous study (40). The samples were processed on an AU480 Clinical Chemistry Analyser (Beckman Coulter Australia Pty Ltd, Lane Cove West, NSW) to analyze alanine transaminase (ALT), total bilirubin (TB), urea, creatinine, cholesterol, and total protein, while Randox kits (Randox Australia Pty Ltd, Parramatta, NSW) were used for glutamate dehydrogenase (GLDH). Each sample was analyzed in triplicate, and the results were expressed as an average.

### Serum Immune Response

Serum lysozyme activity for both pre- and post-challenge was determined for six fish/treatment (two fish/replicate) following the turbidimetric method described by Le and Fotedar (41) and Bowden et al. (42). Briefly, 50 μl of each serum, repeated in triplicate, was pipetted in a 96-well-plate (Iwaki, Tokyo, Japan), and then 50 μl of *Micrococcus lysodeiktikus* suspended in PBS (0.25 mg/ml) (Sigma-Aldrich, St. Louis, MO, USA) was added into each well. The absorbance was measured using an MS212 reader (Titertek Plus, Tecan, Austria) at 450 nm every 2 min, for a total of 20 min at 25°C. One unit of lysozyme activity was defined as the amount of enzyme resulting in a decrease in absorbance of 0.001/min. The results are presented as U/ml.

Serum bactericidal activity for both before and after challenge was determined for six fish/treatment (two fish/replicate) following the protocol of Ueda et al. (43) and Le and Fotedar (41). Fifty microliters of *Vibrio anguillarum*, obtained from the Department of Agriculture and Food, Perth, WA, Australia, suspended in phosphate-buffered saline (0.1 M, pH 7.2) was added to 50 μl serum, and the same volume of bacterial suspension was added to 50 μl of PBS as control. The mixture was then incubated for 30 min at 25°C. Following the reaction, 50 μl from the mixture was plated onto triplicated tryptone soya agar and incubated for 24 h at 25°C. Bactericidal activity was calculated as a decrease in the number of viable *V. anguillarum* cells, which is log10 colony-forming units (CFU)/ml in the control minus log10 CFU/ml in serum.

### RNA Extraction and Gene Expression Analysis

Six fish per treatment (two/replicate) were euthanized using 175 mg l^−1^ AQUI-S®, and the liver, spleen, and head kidney were excised 42 days post-feeding and also 24-h post-challenge. The samples were immediately preserved in RNA Later (Sigma-Aldrich, Germany) and then stored at −80°C till extraction of RNA. Total RNA was extracted using 1 ml Trizol TM reagent (Invitrogen) based on the manufacturer's protocols from ~50–100 mg of various tissue samples. The degradation and contamination of RNA were checked by gel electrophoresis, and the purity of RNA was monitored on a NanoDrop spectrophotometer 2000c (Thermo Fisher Scientific, USA). cDNA synthesis was carried out from 1 μg of total RNA by Omnicript RT kit (Qiagen, Hilden, Germany) as per the protocols of the manufacturing company. The specific primers of selected genes and reference genes used were as from the earlier published studies. RT-qPCR using PowerUp™ Cyber Green Master Mix (Thermo Scientific, USA) with 7500 Real-Time PCR System (Applied Biosystems, USA) was conducted as described in our earlier study. The relative expression of target genes was normalized to the *18S rRNA* and *Ef1-a*, housekeeping genes (Table 3), and calculated using the 2^−ΔΔct^ method.

**Table 3:** Primer sequence of heat shock related gene and immune related cytokines.

**Primer**	**Forward primer (5^**′**^-3^**′**^)**	**Reverse primer (5^**′**^-3^**′**^)**	**References**
HSP90	ACCTCCCTCACAGAATACC	CTCTTGCCATCAAACTCC	(44)
IL-1β	ACAACGTCATGGAGCTCTGG	TCTTTGTCCTTCACCGCCTC	(45)
IL-10	CGACCAGCTCAAGAGTGATG	AGAGGCTGCATGGTTTCTGT	(45)
18S rRNA	TGGTTAATTCCGATAACGAACGA	CGCCACTTGTCCCTCTAAGAA	(44)
Ef1-a	AAATTGGCGGTATTGGAAC	GGGAGCAAAGGTGACGAC	(44)

### Calculation and Statistical Analysis

Growth performance in terms of weight gain, specific growth rate, feed intake (FI), feed conversion (FCR), and biometry indices in terms of condition factor, hepatosomatic index, and intraperitoneal fat index (IFI) were calculated as follows:

$$\begin{array}{l} \text{Weight gain }(\text{ g})\;=\;[(\text{mean final weight}-\text{mean initial weight})\\ /(\text{mean initial weight})]\\ \text{Specific growth rate }(\text{\%day})=\;[(\text{ln }(\text{final body weight})\\ -\text{ln }(\text{pooled initial weight}))/\text{days}]\;\times 100\\ \text{Feed intake }(\text{g}/\text{fish da}{\text{y}}_{-1})=[(\text{dry diet given}-\text{dry}\\ \text{remaining diet recovered})/\text{days of experiment})/\text{no}.\text{ of fish}]\\ \text{Feed conversion ratio}=\;[(\text{dry feed fed})/(\text{wet weight gain})]\\ \text{Condition factor }(\text{\%})=\;[\text{final body weight }(\text{g})\\ /\text{body length c}{\text{m}}^{3})]\;\times 100\\ \text{Hepatosomatic index }(\text{\%})=\;[\text{liver weight }(\text{g})\\ /\text{whole body weight }(\text{g})]\times 100\\ \text{Intraperitoneal fat index }\left(\text{\%}\right)\;=\;[\text{Intraperitoneal fat}\\ \text{weight }(\text{g})/\text{whole body weight }(\text{g})]\;\times 100 \end{array}$$

The filet lipid quality of barramundi fed control and HI-larvae-supplemented PBM diets was determined using two important lipid indexes, atherogenicity (AI) and thrombogenicity (TI), as follows:

$$\begin{array}{l} \text{AI}=\;\left(aC12:0+bC14:0+c16:0\right)/\left(dP+eM+fM^{\prime }\right) \end{array}$$

where *P* is the sum of n3 and n6 polyunsaturated fatty acids (PUFA), *M* is oleic acid, and *M*′ is the sum of other monounsaturated fatty acids (MUFA); *a, b, c, d, e*, and *f* are empirical constants, with *b* = 4 and the other constants = 1.

$$\begin{array}{l} \text{TI}=\;\left(\text{C}14:0+\text{C}16:0+\text{C}18:0\right)/[\left(\text{nM}+\text{n}{\text{M}}^{\prime }+\text{p}(\text{n}6\right)\\ \;+\text{q }\left(\text{n}3\right)+(\text{n}3/6)] \end{array}$$

where M and M′ are as before and n, o, p, and q are empirical constants, with n, o, p = 0.5 and q = 3.

Unless specified otherwise, all results are presented as mean ± SE. All data were subjected to Shapiro–Wilk's and Levene's tests to test the normal distribution and homogeneity of variances. One-way ANOVA, followed by Dunnett's multiple-comparisons test, was performed on growth performance, biometry indices, heat shock-related gene expression, and adipocyte cell size to test the significant differences between the experimental groups. The survival of 42 days post-feeding groups and 14 days post-challenge groups was compared by the Kaplan–Meier method based on the pairwise multiple-comparison log-rank (Mantel–Cox) test. Data on serum biochemistry, cytokine expression, and skin histomorphology after the challenge test were subjected to a two-way ANOVA using the general linear models procedure where “diet” and “challenge” were used as the main factors. Data from the pre- and post-challenge tests were compared by paired *t*-test.

## Results

### Fish Performance and Feed Utilization

**Figure 1:**
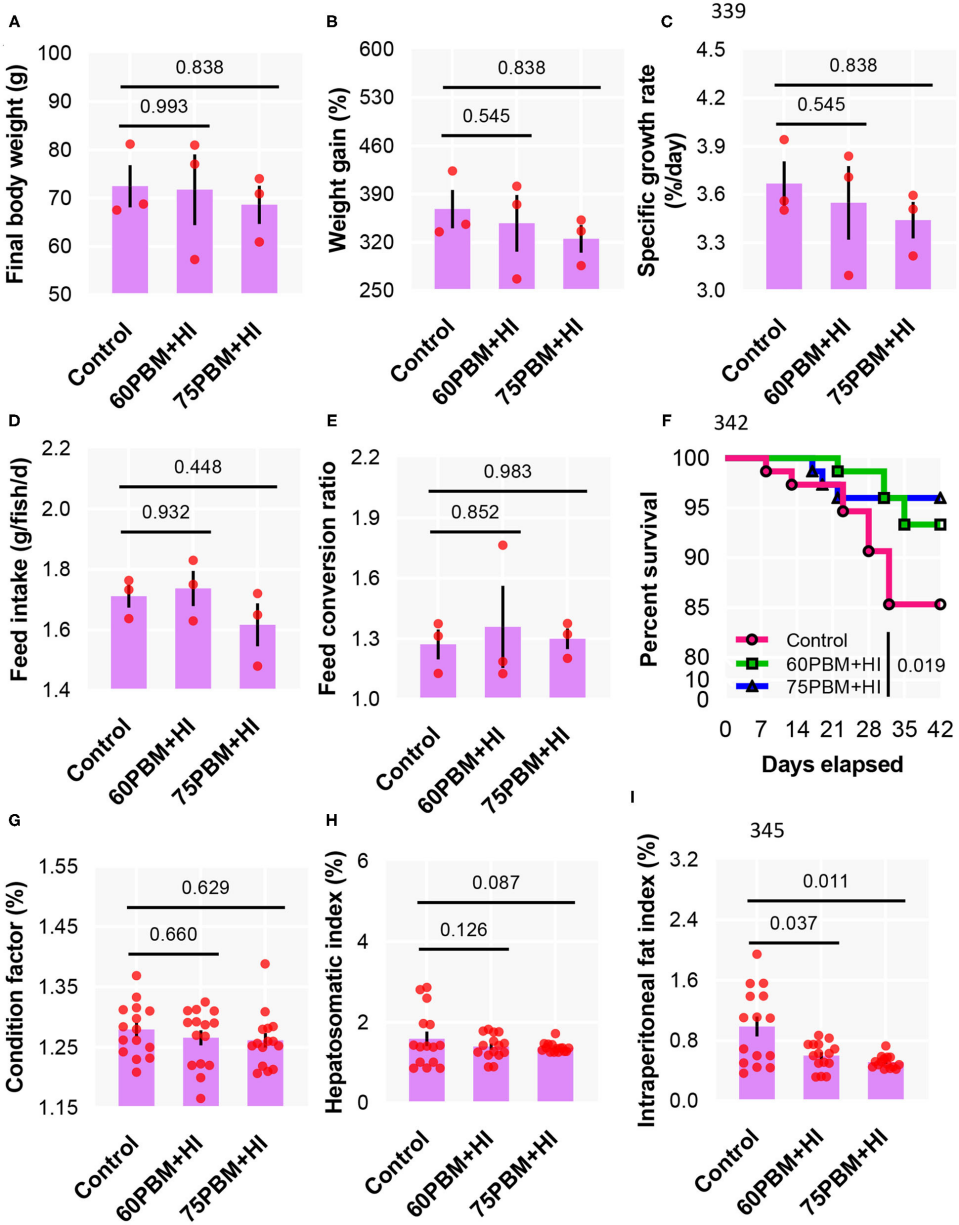
Growth performance **(A–C)**, feed utilization **(D,E)**, survival **(F)**, and biometry indices **(G–I)** of barramundi 6 weeks post-feeding with control, 60% FM replacement diet (60PBM + HI), and 75% FM replacement diet (75PBM + HI), supplemented with 10% full-fat HI larvae. The results represent mean ± SEM of three values. *P*-values on the top of the bar with scatter dot plot denote significant differences between control vs. 60PBM + HI-fed and 75PBM + HI-fed fish (ordinary one-way ANOVA, followed by Dunnet's multiple comparison test, *P* < 0.05). The asterisk denotes significant differences between control vs. 60PBM + HI-fed and 75PBM + HI-fed fish (Kaplan–Meier survival method, followed by log-rank test, *P* < 0.05).

The weight of barramundi at the termination of 6 week of growth increased more than 4-fold when compared to the initial weight, with no significant variations in growth performance as measured by final body weight (Figure 1A), weight gain (Figure 1B), specific growth rate (Figure 1C), FI (Figure 1D), and FCR (Figure 1E) between the three test diets. At the end of the trial, the 60PBM + HI- and 75PBM + HI-fed groups showed a significantly higher survival rate than the control (Figure 1F). None of the test diets had significant effects on the biometry indices, including condition factor (Figure 1G) and hepatosomatic index (Figure 1H). However, the IFI decreased significantly in 60PBM + HI- and 75PBM + HI-fed groups when compared with the control (Figure 1I).

### Filet Fatty Acid Composition

The fatty acid profile of the barramundi filets at 6 weeks post-feeding with the experimental diets is presented in Table 4. Saturated fatty acid (SFA) content varied among dietary groups, increasing significantly from control to 60PBM + HI and 75PBM + HI. The majority of the individual SFAs, with the exception of C15:0 and C17:0, increased significantly in HI-supplemented PBM diets when compared to the control. The majority of individual MUFAs were affected by diets, with most showing a significant increase in HI-supplemented PBM diets compared to control. HI-supplemented PBM diets showed an increased content of PUFA, particularly α-calendic acid (C18:3n6), alpha-linolenic acid (C18:3n3), stearidonic acid (C18:4n3), dihomo-gamma-linolenic acid (C20:3n6), arachidonic acid (C20:4n6), and eicosatrienoic acid (C20:3n3), while adrenic acid (C22:4n6) and docosahexaenoic acid (C22:6n3) declined significantly in HI-supplemented diets when compared with the control. Regarding lipid quality, both AI and TI increased in fish fed 60PBM + HI and 75PBM + HI.

**Table 4:** Filet fatty acid (mg/100 g on dry matter basis) composition of barramundi fed control and HI-larvae supplemented diets at the termination of the 6-week trial.

	**Control**	**60PBM + HI**	**75PBM + HI**	**ANOVA *P***
C10:0	0.78 ± 0.03^b^	4.81 ± 0.72^a^	4.31 ± 0.06^a^	0.00
C12:0	1.02 ± 0.09^b^	295.03 ± 33.71^a^	294.63 ± 2.47^a^	0.00
C13:0	0.60 ± 0.00^b^	1.02 ± 0.12^ab^	0.92 ± 0.00^a^	0.01
C14:0	67.46 ± 0.77^b^	217.78 ± 21.68^a^	217.70 ± 1.96^a^	0.00
C14:1n5	0.97 ± 0.03^b^	6.43 ± 0.73^a^	6.73 ± 0.07^a^	0.00
C15:0	17.76 ± 0.63	19.04 ± 1.78	17.81 ± 0.21	0.67
C15:1	0.00 ± 0.00	0.00 ± 0.00	0.18 ± 0.20	0.42
C16:0	713.65 ± 10.19^b^	1,253.87 ± 105.68^a^	1,319.91 ± 13.68^a^	0.00
C16:1n7	130.78 ± 1.90^b^	281.81 ± 27.55^a^	287.55 ± 2.38^a^	0.00
C17:0	44.02 ± 0.52	46.25 ± 3.97	41.62 ± 0.82	0.43
C17:1	22.70 ± 0.31	27.71 ± 2.80	23.65 ± 1.08	0.18
C18:0	278.21 ± 4.07^b^	427.36 ± 27.76^a^	452.18 ± 5.83^a^	0.00
C18:1cis + trans	859.76 ± 5.73^b^	2,698.63 ± 240.32^a^	2,974.63 ± 63.88^a^	0.00
C18:2 trans	17.50 ± 15.25	7.06 ± 1.52	7.05 ± 0.67	0.65
C18:2 cis	320.82 ± 3.44^b^	1,169.13 ± 101.94^a^	1,229.36 ± 17.46^a^	0.00
C18:3n6	17.51 ± 1.42^c^	48.60 ± 0.80^b^	58.10 ± 1.30^a^	0.00
C18:3n3	55.12 ± 0.38^b^	215.13 ± 19.68^a^	216.91 ± 1.96^a^	0.00
C18:4n3#	17.01 ± 1.06^b^	44.59 ± 2.66^a^	44.67 ± 1.21^a^	0.00
C20:0	8.95 ± 0.22^b^	17.81 ± 1.00^a^	18.59 ± 0.21^a^	0.00
C20:1	36.62 ± 0.38^b^	119.40 ± 10.53^a^	115.02 ± 0.97^a^	0.00
C20:2	8.84 ± 0.03^b^	16.73 ± 1.26^a^	17.02 ± 0.26^a^	0.00
C21:0	4.05 ± 0.18^b^	6.64 ± 0.45^a^	6.78 ± 0.15^a^	0.00
C20:3n6	25.68 ± 0.57^b^	46.67 ± 3.26^a^	52.49 ± 0.53^a^	0.00
C20:4n6	92.29 ± 1.90^b^	121.96 ± 6.52^a^	120.73 ± 3.17^a^	0.00
C20:3n3	4.61 ± 0.09^b^	6.83 ± 0.52^a^	6.77 ± 0.33^a^	0.01
C22:0	3.58 ± 0.03^b^	7.50 ± 0.46^a^	7.66 ± 0.27^a^	0.00
C20:5n3	109.31 ± 1.78	126.12 ± 9.98	113.69 ± 1.31	0.19
C22:1n9	4.03 ± 0.03^b^	11.61 ± 1.04^a^	11.85 ± 0.09^a^	0.00
C22:2	0.00 ± 0.00^b^	0.93 ± 0.09^a^	1.00 ± 0.00^a^	0.00
C23:0	13.39 ± 0.68^b^	25.64 ± 1.36^a^	27.84 ± 0.15^a^	0.00
C22:4n6#	62.67 ± 1.07^a^	29.39 ± 1.68^b^	23.40 ± 0.59^c^	0.00
C22:5n3#	71.50 ± 0.95	82.68 ± 5.44	79.49 ± 1.05	0.12
C24:1	11.81 ± 0.12^b^	14.76 ± 0.91^a^	14.72 ± 0.17^a^	0.01
C22:6n3	683.13 ± 12.43^a^	447.58 ± 23.47^b^	377.51 ± 8.64^c^	0.00
∑SFA	1,153.45 ± 15.14^a^	2,322.75 ± 197.52^a^	2,409.95 ± 21.74^a^	0.00
∑MUFA	1,066.66 ± 8.25^b^	3,160.35 ± 283.49^a^	3,434.32 ± 67.55^a^	0.00
∑PUFA	1,485.98 ± 24.56^b^	2,363.40 ± 174.89^a^	2,348.18 ± 34.04^a^	0.00
∑n-3	940.67 ± 15.92	922.93 ± 61.54	839.03 ± 11.05	0.20
∑n-6	198.15 ± 3.87^b^	246.63 ± 12.20^a^	254.72 ± 4.82^a^	0.01
∑n-3/∑n-6	4.75 ± 0.03^a^	3.74 ± 0.15^b^	3.29 ± 0.00^c^	0.00
Atherogenicity	0.32 ± 0.00^b^	0.34 ± 0.00^a^	0.33 ± 0.00^ab^	0.01
Thrombogenicity	0.27 ± 0.00^c^	0.33 ± 0.00^b^	0.34 ± 0.00^a^	0.00

*Different superscript letters within the same row denote significant differences between control vs. 60PBM + HI-fed and 75PBM + HI-fed fish (ordinary one-way ANOVA, followed by Dunnett's multiple-comparisons test, P < 0.05)*.

*HI, Hermetia illucens; PBM, poultry by-product meal; SFA, saturated fatty acids; MUFA, monounsaturated fatty acids; PUFA, polyunsaturated fatty acids*.

### Histological Analysis and Expression of HSP90 in the Liver

**Figure 2:**
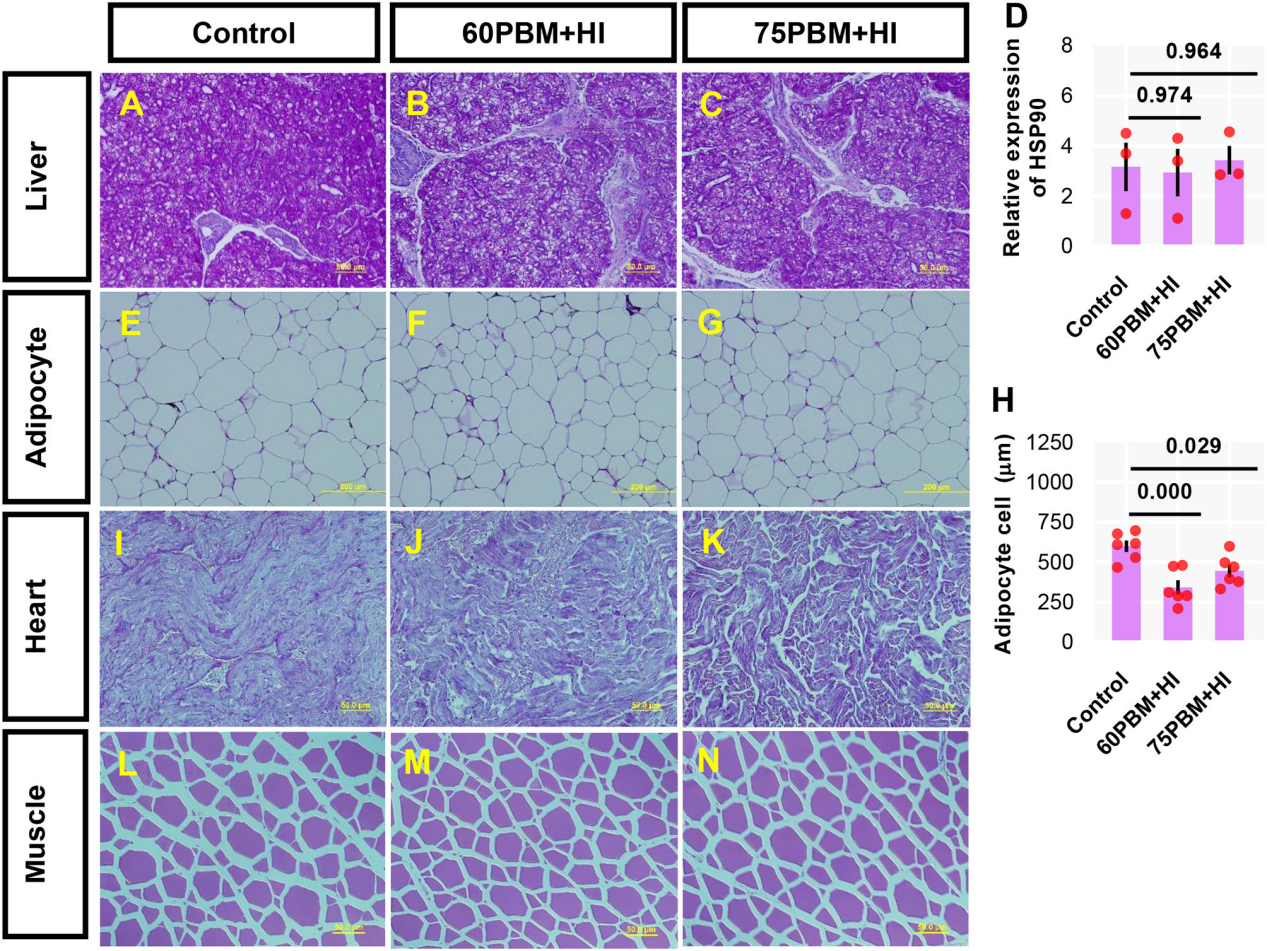
Light microscopy of the liver **(A–C)** [periodic acid–Schiff (PAS) stain, ×40 magnification), adipose cell size **(E–G)** (PAS stain, ×20 magnification), heart **(I–K)** (PAS stain, ×40 magnification), and muscle **(L–N)** (PAS stain, ×40 magnification) of juvenile barramundi 6 weeks post-feeding with control, 60% FM replacement diet (60PBM + HI) and 75% FM replacement diet (75PBM + HI), supplemented with 10% full-fat HI larvae. **(D,H)** Variation in the expression of hepatic HSP90 (*n* = 3) and adipocyte cell size (*n* = 6) in response to different levels of PBM supplemented with HI. The results represent mean ± SEM. *P*-values on the top of the bar with scatter dot plot denote significant differences between control vs. 60PBM + HI-fed and 75PBM + HI-fed fish (ordinary one-way ANOVA, followed by Dunnet's multiple comparison test, *P* < 0.05).

The internal architecture of the liver, intraperitoneal fatty tissue, heart, and muscle tissue was evaluated by histology, and the results varied among test groups (Figures 2A–K). The liver of fish fed control and test diets showed no histopathological changes, with a higher amount of glycogen that was characterized by a highly pigmented hepatic cytoplasm (Figures 2A–C). Relative expression of heat shock protein-90 (HSP90) was not induced by the control and other test diets (Figure 2D). All adipocyte sizes in the intraperitoneal fatty tissue (Figures 2E–G), in particular, HI-larvae-supplemented groups, showed a smaller size of adipocyte cells (Figure 2H). The heart (Figures 2I–K) and muscle (Figures 2L–N) tissue results showed no significant difference between the test groups.

### Resistance to *V. harveyi*

**Figure 3:**
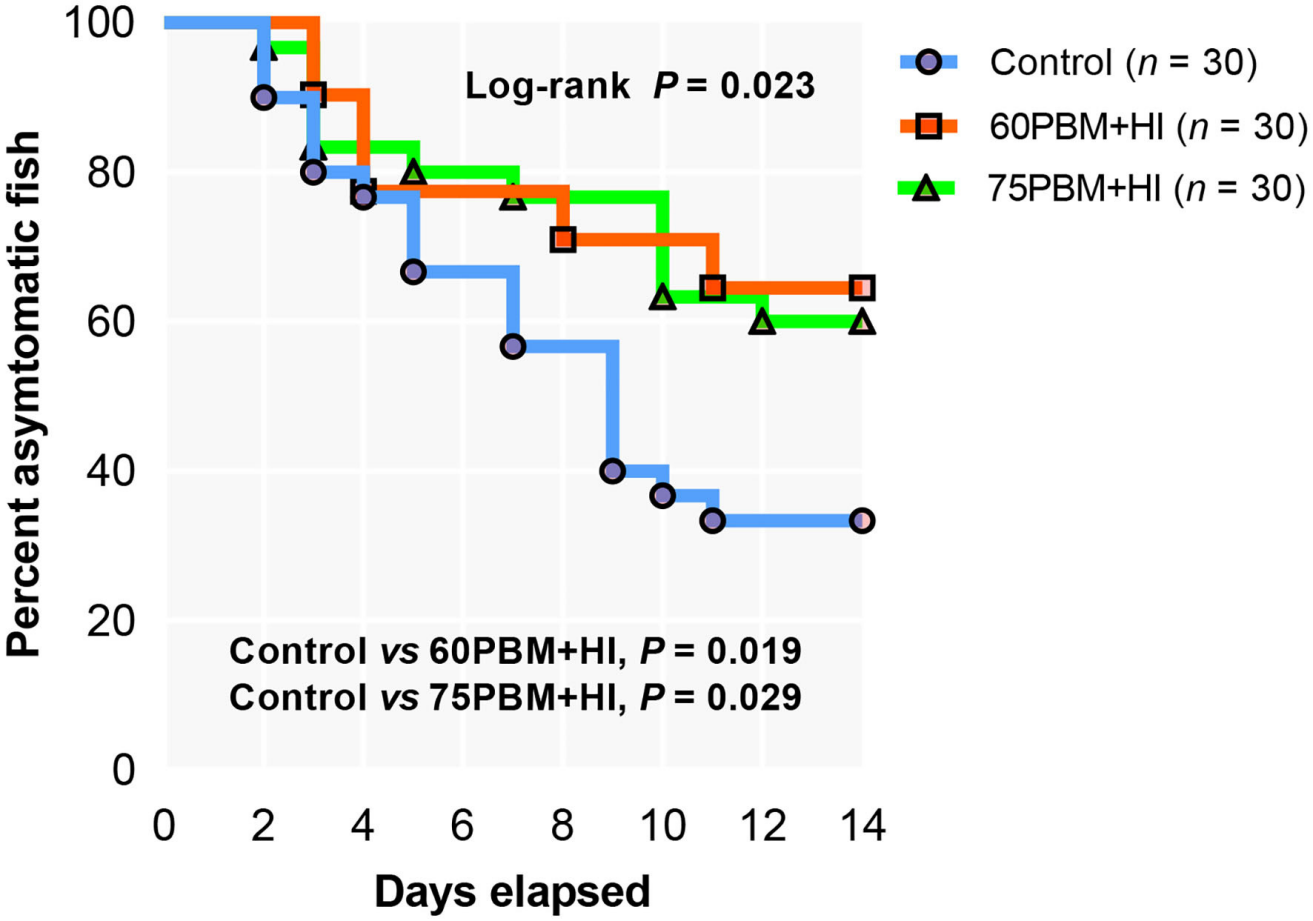
Kaplan–Meier curve of barramundi fed with control, 60% FM replacement diet (60PBM + HI), and 75% FM replacement diet (75PBM + HI), supplemented with 10% full-fat HI larvae in response to *Vibrio harveyi* challenge over a period of 2 weeks. The control groups demonstrated infection at 2 days post-challenge, whereas 60PBM + HI and 74PBM + HI groups demonstrated infection 3 days post-challenge. *P*-values denote significant differences between control vs. 60PBM + HI-fed and 75PBM + HI-fed fish (Kaplan–Meier survival method, followed by log-rank test, *P* < 0.05).

Supplementation of HI larvae with two different levels of PBM significantly modulated the infection rate, showing 64.52 and 60.00% asymptomatic fish in 60PBM + HI and 75PBM + HI, respectively, when compared to 33.33% asymptomatic fish for the control-fed groups (Figure 3).

### Serum Biochemistry

**Figure 4:**
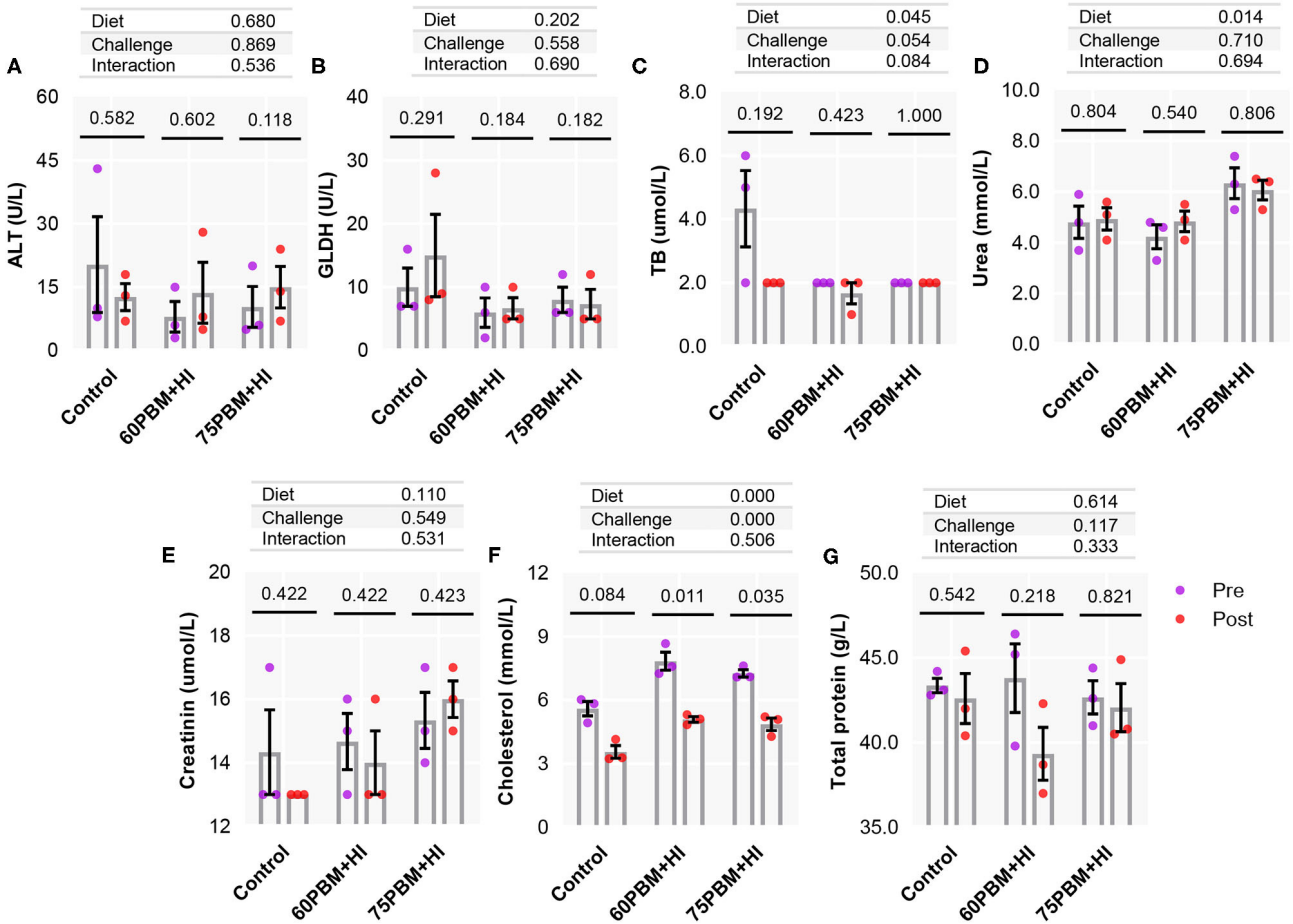
Serum biochemical changes **(A–G)** in pre-challenge and 24-h post-challenge juvenile barramundi fed control, 60% FM replacement diet (60PBM + HI), and 75% FM replacement diet (75PBM + HI), supplemented with 10% full-fat HI larvae. The bar represents the mean of three technical replicates. The violet and the red markers (each marker represents the mean of two biological replicates) denote pre-challenge and post-challenge groups, respectively. *P*-values on the top of the bar with scatter dot plot denote significant differences between pre- and post-challenge groups fed control and HI-supplemented PBM diets (paired *t*-test, *P* < 0.05). The effect of diet and challenge and their interaction were analyzed by two-way ANOVA with Dunnett's multiple-comparisons test.

Considering serum biochemical parameters (Figures 4A–G), the factors “diet and challenge” had no significant effects on all parameters, with the exception of TB and cholesterol. Two-way ANOVA analysis demonstrated that diet had a significant effect on TB, as manifested by a significantly lower level of TB in HI-larvae-supplemented PBM diets compared to control (*P* < 0.05). Meanwhile, as reported in Figure 4F, a significant effect with “diet and challenge” was only reported for cholesterol, which decreased in post-challenge 60PBM + HI and 75PBM + HI groups (*P* < 0.001) compared to the pre-challenge groups, as revealed by paired *t*-test. In both before and after the challenge test, cholesterol was significantly higher in HI-supplemented PBM diets. Additionally, no significant interaction between “diet” and “challenge” was observed for all parameters (*P* > 0.05).

### Skin Mucosal Response

**Figure 5:**
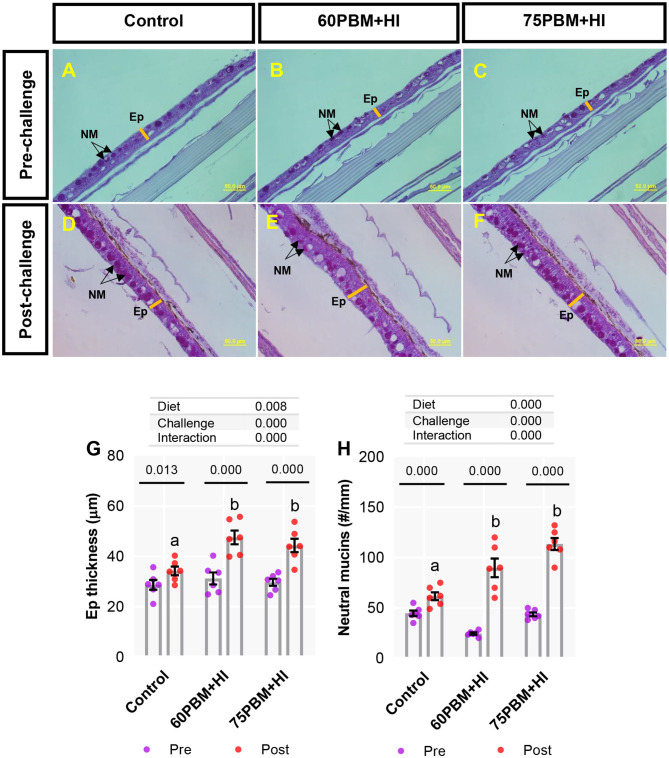
Light microscopy of skin histometry and histochemistry (periodic acid–Schiff, ×40 magnification) in pre-challenge **(A–C)** and at 24-h post-challenge **(D–F)** barramundi fed control, 60% FM replacement diet (60PBM + HI), and 75% FM replacement diet (75PBM + HI), supplemented with 10% full-fat HI larvae. Variation in the Ep thickness **(G)** and the number of goblet cells producing neutral mucins **(H)** in the skin of pre-challenge and post-challenge barramundi fed different test diets. *P*-values on the top of the bar with scatter dot plot denote significant differences between pre- and post-challenge groups fed control and HI-supplemented PBM diets (paired *t*-test, *P* < 0.05). Different letters on the top of the bar with scatter dot plot denote significant differences between control vs. 60PBM + HI-fed and 75PBM + HI-fed fish (one-way ANOVA, followed by Dunnett's multiple-comparisons test, *P* < 0.05). The effect of “diet” and “challenge” and their interaction were analyzed by two-way ANOVA with Dunnett's multiple-comparisons test. The bar indicates the mean of six values. The violet and the red markers denote pre-challenge and post-challenge groups, respectively. *EP*, epidermis; NM, neutral mucins.

Skin epidermis thickness (Ep) and neutral mucins (NM) in pre-challenge and at 24-h post-challenge groups are presented in Figures 5A–F. Two-way ANOVA demonstrated that both “diet” and “challenge” had significant effects on Ep thickness and NM, and a significant interaction was also observed between the factors. As reported in Figures 5G,H, both Ep and NM, in response to 24-h post-challenge with *V. harveyi*, significantly increased in all test diet groups when compared with all pre-challenge groups (paired *t*-test, *P* < 0.01 and 0.001). Considering the 24-h post-challenge groups, both Ep thickness and NM numbers increased significantly in fish fed with HI-supplemented PBM than the fish fed a control diet.

### Serum Immunity and Cytokine Expression

**Figure 6:**
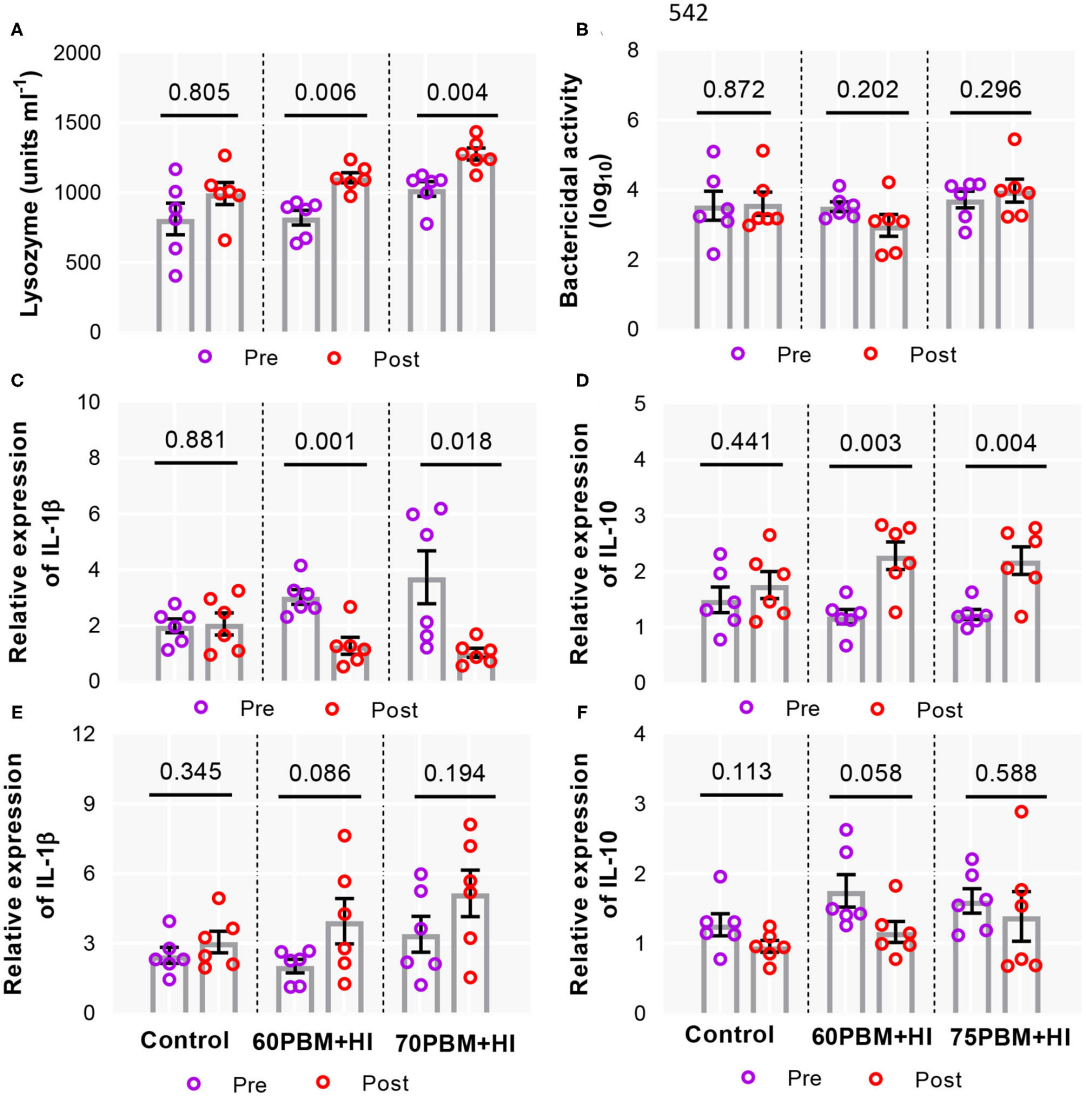
Immune response in serum and cytokine expression participated in the inflammatory response in barramundi head kidney and spleen. Serum lysozyme **(A)** and bactericidal activity **(B)** as well as qPCR analysis of IL-1β and IL-10 both in the head kidney **(C,D)** and spleen **(E,F)** isolated from barramundi pre-challenge control, 60PBM + HI and 75PBM + HI and 24-h post-challenge with *Vibrio harveyi*. *P*-values on the top of the scatter dot plot denote significant differences between pre- and post-challenge groups fed control and HI-supplemented PBM diets (paired *t*-test, *P* < 0.05). Lower standard error in the bar indicates the adequacy of using minimum biological replicates for serum immunological assessment.

Serum immune response, including lysozyme and bactericidal activity and cytokine expression, including interleukin (1β) IL-1β and IL-10 in head kidney and spleen in response to diets and challenge, are presented in Figure 6, Table 5. After 24 h of bacterial infection, serum lysozyme increased significantly in post-challenge groups fed HI-supplemented diets (Figure 6A) when compared with the pre-challenge groups, whereas dietary treatments and bacterial challenge had no effects on bactericidal activity (Figure 6B). Meanwhile, irrespective of the experimental diets, IL-1β expression level in the head kidney of 24-h post-challenge groups fed HI-supplemented diets were down-regulated with respect to pre-challenge groups (Figure 6C). These groups showed an upregulation of IL-10 (Figure 6D). However, there were no significant effects on the expression level of IL-1β and IL-10 in response to 24-h post-challenge in the spleen of barramundi fed control and HI-supplemented diets (Figures 6E,F).

**Table 5:** Factorial analysis on the effect of test diets and challenge time and their interaction on serum immune response (lysozyme and bactericidal activity) and cytokine expression (IL-1β and IL-10) both in the kidney and spleen.

	**Factors**		**Interaction**
**Parameters**	**diet**	**Challenge**	**diet × challenge**
Serum lysozyme	0.061	0.003	0.218
Serum bactericidal activity	0.164	0.774	0.418
IL-1β (kidney)	0.749	0.001	0.118
IL-10 (kidney)	0.850	0.001	0.119
IL-1β (spleen)	0.086	0.019	0.578
IL-10 (Spleen)	0.149	0.037	0.665

## Discussion

The present study incorporated 60PBM + HI and 75PBM + HI, and neither diet had significant effects on growth performance when compared to the control. Similarly, many previous studies have reported that the dietary inclusion of HI larvae meal did not influence the growth performance of fish, including Jian carp, *Cyprinus carpio* var. Jian (46), European seabass, *Dicentrarchus labrax* (47), Atlantic salmon, *Salmo salar* (48), clownfish (49), and rainbow trout, *Oncorhynchus mykiss* Walbaum (50). However, it was also previously found that 45PBM + HI significantly influenced the growth performance, intestinal mucosal immunity, and resistance to *V. harveyi* compared to FM-based diet, while 90PBM + HI negatively impacted the health of barramundi (37). Similar to Belforti et al. (51), HI inclusion increased the survival, probably meeting the nutritional demands of the barramundi better than the control diet. The amelioration of survival rate might be due to the presence of immunomodulating components, including chitin and antimicrobial peptide, in HI larvae. However, 15% of mortalities could be contributed to cannibalism as some partly eaten dead fish were found in the control treatment, similar to our previous studies (13, 40).

Fatty acid composition in a diet has been reported to influence the fish muscle fatty acid composition, which is an important trait for consumers since some fatty acids, in particular, MUFA and PUFA, promote health (50). Dietary inclusion of PBM to replace FM previously was reported to modify the muscle fatty acids composition of fish (13). In this study, high levels of lauric acid (C12:0), myristic acid (C14:0), and palmitic acid (C16:0) resulted in a higher total SFA content in the muscle of HI-supplemented-PBM-fed fish, possibly owing to the consequence of the high abundance of C12:0, C14:0, and C16:0 in these respective diets. Similarly, feeding HI larvae meal-based diet increased total SFA due to a higher proportion of lauric acid in rainbow trout [*O. mykiss*; (50)], Jian carp [*Cyprinus carpio* var. Jian; (46)], and Atlantic salmon [*Salmo salar*; (52, 53)]. In addition, a PBM-based diet was reported to elevate the total SFA in juvenile black sea bass as a consequence of a high abundance of palmitic acid (54). The relatively lower levels of lauric acid in the muscle of HI-larvae-supplemented-PBM-fed fish in comparison to those respective diets may suggest that the fatty acid was readily utilized to produce energy by oxidation. However, adding 33, 67, and 100% PBM in the diet of juvenile totoaba, *Totoaba macdonaldi*, decreased the levels of essential fatty acids in the muscle (55), and similar results were reported in barramundi muscle when fed 75 and 100% PBM, either unprocessed or bioprocessed (13). Interestingly, a significantly higher level of MUFA and PUFA in the muscle of barramundi fed HI-larvae-meal-supplemented PBM diets than the control-fed fish was observed in this study. This was mainly due to the presence of a higher proportion of MUFA and PUFA in the respective diets. However, the substitution of FM with partially defatted HI larvae meal decreased the levels of PUFA in the muscle of rainbow trout (50). This heterogeneity might be due to the utilization of different growing substrates for HI larvae culture since the nutritional profile largely depends on the growing substrate. For instance, HI larvae fed with vegetable by-products showed no evidence of eicosapentaenoic acid and docosahexaenoic acid (50) that was present in HI larvae fed carp mince utilized in this study. However, high levels of MUFA and PUFA in fish are associated with lessening the risk of neurological diseases, particularly myocardial infarction and cardiovascular disease. AI and TI are two important lipid indices used to determine the contribution of SFA, MUFA, and PUFA, and respected levels are associated with consumer health. Both the AI and TI values of muscle from barramundi in this study were <1.0 and were significantly increased by two different levels of PBM supplemented with 10% HI larvae, indicating that the fish produced were healthier for human consumption (50).

As documented in many studies (56–60), the dietary substitution of FM with alternative protein ingredients can impact the internal architecture of liver, in particular, increasing hepatic lipid deposition and lipid vacuoles, known as steatosis, in many fish species. The 6-week feeding trial reported here did not impose negative effects on the liver structure, which was further evidenced by the insignificant effect of HI-supplemented PBM diets on the mRNA expression level of the stress-related gene, HSP90, in the liver. In our earlier study, fish fed 45PBM + HI showed no obvious histopathological alteration and upregulation of HSP70 and HSP90 in the liver, while multifocal necrosis and upregulation of HSP70 and HSP90 were found in the liver of fish fed 90PBM + HI (37). However, hepatic vacuoles and lipid droplets, a sign of hepatic steatosis, increased in the liver of hybrid grouper, Epinephelus *fuscoguttatus*♀ × *Epinephelus lanceolatus*♂, when fed gradually increasing levels of an animal protein blend (20–80%), including PBM, shrimp meal, and spray-dried blood meal (61). The fact that there was no histopathological alteration, in the present study, in the liver of fish fed higher levels (up to 75%) of PBM might be due to the presence of chitin in HI larvae, as chitin and its derivatives have been reported to have a significant role in reducing the synthesis of fatty acids as well as boosting the hydrolysis of lipoproteins and triglyceride in the liver of fish and other animals (46, 62, 63).

Feeding female tenches, *Tinca*, over a period of 86 days with graded levels of PBM (25.7–100%) increased the intramuscular fatty tissue deposition (64). In the present study, dietary administration of 60 and 75% PBM, supplemented with 10% HI larvae, in the diet of barramundi resulted in a significant decline in intraperitoneal fat content which could be due to diminished adipose cells as revealed by the histology of the intraperitoneal fatty tissue. Regardless of PBM inclusion, dietary inclusion of HI larvae could influence the intraperitoneal fat content. For instance, a decrease in lipid deposition in the intraperitoneal fat tissue with concomitant upregulation of PPARα, a lipid metabolism-relevant gene, was found in juvenile Jian carp, *Cyprinus carpio* var. Jian fed 75 and 100% of HI larvae oil (65). There are several medium-chain fatty acids (MCFA), particularly C_6_-C_12_ physiologically active fatty acid components in HI larvae, which have been reported to decrease lipid deposition by enhancing the energy availability and reducing the deposition of adipose tissue (65, 66). Similar results in terms of MCFA were observed in the HI-supplemented PBM diets and HI larvae meal in this study. In addition, the presence of chitin in HI larvae could modulate the intraperitoneal fat, as suggested by Hossain and Blair (67) who reported reduced body fat content in broiler chickens in response to the dietary inclusion of chitin. It has also been reported that cardiac steatosis in the heart and necrosis and myodegeneration in the muscle may also occur due to nutritional deficiency (68, 69). In the present study, there were no histopathological changes in heart and muscle tissue, indicating no negative effects of higher inclusion of PBM aligned with HI supplementation.

As reported in earlier studies, feeding 2.5% maggot meal to black carp, *Mylopharyngodon piceus*, modulated the survival rate against *Aeromonas hydrophila* (70), and addition of 5% housefly pupae meal protected by 100% the red sea bream, *Pagrus major*, from *Edwardsiella tarda* infection (71). In this study, the infection rate of barramundi fed HI-supplemented diets was influenced after 14 days of challenge to *V. harveyi*. HI larvae was previously reported to improve the gut health of rainbow trout, *O. mykiss*, by increasing the abundance of *Carnobacterium* genus, which are well-documented probiotics in salmonids with several beneficial effects, including *in vitro* growth inhibition of pathogens and *in vivo* improvement of disease resistance (9). Such modulation of disease resistance following HI supplementation could be explained by the presence of bioactive compounds such as chitin and antimicrobial peptides as well as MCFA, in particular, lauric acid (72). Chitin is reported to hinder the growth of pathogens in fish by boosting the growth of beneficial intestinal bacteria (73), and lauric acid is also reported to be active against bacteria (72). In addition, Elhag et al. (4) and Park et al. (3) extracted low molecular weight antimicrobial peptides exhibiting antibacterial and antifungal activity from HI larvae.

ALT, GLDH, and TB are used as indicators of liver and kidney health in fish, and increased levels may indicate cellular damage, characterized by degeneration, necrosis, and destruction of the liver and kidney. Although there was no significant impact of diets and challenge on the serum ALT and GLDH levels, a decreasing tendency was observed in HI-supplemented diets compared to control. Meanwhile, HI-supplemented diets significantly decreased the TB levels of barramundi. These results may suggest that HI supplementation could hamper the negative effects caused by excessive levels of PBM and protect the liver and kidney from cell damage. Similarly, in this study, no histopathological changes were observed in the liver as determined by histological evaluation. Conversely, it was previously reported that feeding barramundi with PBM (406 and 300 g/kg), replacing FM, hampered the liver function by increasing plasma ALT and GLDH (74). The dietary inclusion of APB likewise impaired the liver health of hybrid grouper by increasing the level of ALT and AST (61). The heterogeneity between those studies and the current findings could be due to the presence of chitin or other functional components such as antimicrobial peptide and/or bioactive polysaccharides in HI larvae meal (37). Chitosan existed in the chitin of HI larvae meal, containing cholesterol-lowering properties (known as hypocholesterolemia) in fish (47, 75). These polymers have been shown to have hypocholesterolemic effects by binding with lipid (cholesterol) micelles, hindering their absorption, elevating bile acid secretion, and interfering with normal lipid digestion and absorption in the intestinal tract as well as biosynthesis of fatty acids in hepatocytes (8, 76–78). Hypocholesterolemic effects were found in European sea bass, *Dicentrarchus labrax*, fed 6.5–19.5% HI pre-pupae meal (47), juvenile mandarin fish, *Siniperca scherzeri*, fed 30% yellow mealworm (79), and Jian carp, *Cyprinus carpio* var. Jian, fed 68–90% silkworm pupae (80); however, such effects were not observed in the present study. Both in pre-challenge and post-challenge conditions, cholesterol level increased in HI-supplemented-PBM-fed barramundi, suggesting that barramundi could have chitinase activity, which remains to be studied. Moreover, cholesterol levels in diets may influence the level of cholesterol in fish (46). The full-fat HI larvae meal used in this study may contain more cholesterol than FM, therefore resulting in higher cholesterol levels in the HI-supplemented-PBM-fed barramundi. However, the cholesterol levels of fish fed any of the test diets were within the normal range for barramundi. Interestingly, the cholesterol levels in the serum of post-challenge HI-supplemented groups had significantly lower levels than the pre-challenge HI-supplemented groups, demonstrating the hypocholesterolemic effects of the challenge on barramundi. A similar effect was observed previously in 24 and 72-h post-challenge barramundi in response to *V. harveyi* infection (14, 40). Food deprivation has been reported to affect the fish's serum cholesterol, which is usually compensated from the body reserves during fasting (81, 82). Hence, lower cholesterol in post-challenge fish could be hypothesized as due to the lower feed supply during the challenge condition (14).

Skin in fish constitutes a large relative surface area when compared to other vertebrates, perhaps due to the fact that there is continuous contact with a variety of unfavorable biotic and abiotic hazards (39). Generally, infectious agents start the infection process in the mucus surface (83), leading to the production of goblet/mucus cells. The goblet cell densities in the skin are influenced by diet and stressors such as bacteria, viruses, and parasites, and therefore the enumeration of the skin goblet cells can be used to monitor the stress level in fish (84). Moreover, goblet cells in the skin epidermis secrete mucus, which serves as a repository for a variety of biologically active substances and numerous defensive molecules which have been reported to exert an important role in both the innate and the acquired immune systems in fish (85–87). In this study, both the thickness of the epidermis and the number of neutral mucins produced by goblet cells in the skin of post-challenge barramundi fed HI-supplemented PBM diets increased significantly. In comparison between pre- and post-challenge, the thickness of the epidermis and the number of goblet cells increased in all post-challenge groups, which suggests that HI larvae supplementation with PBM could boost skin barrier functions in barramundi. No other studies have been conducted on the skin-associated neutral mucins of fish when fed HI larvae meal to allow a comparison with our present findings. However, HI larvae supplementation with 45PBM significantly influenced the barramundi gut-associated acidic mucins in our previous study (37) and also resulted in a general increase in the number of mucus cells in the intestine of zebrafish, *Danio rerio*, when fed with a diet containing 100% HI larvae meal (88).

In teleost fish, serum lysozyme is an indicator of immune response, protecting fish from infectious disease due to elevated levels decomposing the cell wall of Gram-positive and Gram-negative bacteria (37, 89). Dietary inclusion of 31.9% HI larvae and 10% of the yellow mealworm, *Tenebrio molitor*, elevated the lysozyme activity in the serum of yellow catfish, *Pelteobagrus fulvidraco* (90), and European sea bass, *Dicentrarchus labrax* (91), respectively. In our earlier study, barramundi fed 45% PBM supplemented with 10% HI larvae showed elevated serum lysozyme activity at 24-h post-challenge with *V. harveyi* (37). In line with the previous study, lysozyme activity in response to the 24-h post-challenge with *V. harveyi* reported here increased significantly in barramundi fed 60PBM + HI and 75PBM + HI compared to the pre-challenge groups. The present findings contradict the results of Ye et al. (61), in which increasing levels of animal protein blend impacted the immune function of hybrid grouper, *Epinephelus fuscoguttatus*♀ × *Epinephelus lanceolatus*♂, by inducing the inflammatory response through upregulating the expression level of inflammatory cytokines, particularly IL-8 and IL-10. Although supplementation of 10% HI larvae with 45%PBM modulated the bactericidal activity in 24-h post-challenge groups in our earlier study (37), the bacterial challenge had no significant effect on bactericidal activity in this study. The elevated lysozyme activity in HI-larvae-supplemented groups might be due to the immunomodulating capacity of antibacterial peptides, chitin, and other components found in HI larvae (71, 91–93).

In teleost fish, the head kidney is an important immune organ participating in a wide array of functions, including antigen processing (94) and the phagocytosis process (95). The spleen is another large blood-filtering organ involved in trapping and processing antigens (96). Hence, maintaining immune homeostasis in the head kidney and spleen is important in fish nutrition. Interleukin 1β, an immune-relevant proinflammatory cytokine, is expressed early, following microbial invasion, and can stimulate the immune responses by enhancing different cellular responses such as phagocytosis, chemotaxis, and lysozyme synthesis (97, 98). The expression of IL-1β is regulated by the expression of anti-inflammatory cytokines, including IL-10 (99). Apart from the beneficial effects of HI-larvae-supplemented diet on serum immunity, the relative expression of IL-1β in post-challenge groups fed 60PBM + HI and 75PBM + HI decreased significantly compared to that in pre-challenge groups. In comparison to our findings, pro-inflammatory cytokines (IL-1β, IL-8, and TNF-α) and anti-inflammatory cytokines (*IL-10*) responded strongly to bacteria mimic in the head kidney leukocytes isolated from Atlantic salmon, *Salmo salar*, fed diets containing 66 and 100% of HI larvae meal (100). In considering anti-inflammatory cytokines in this study, up-regulation in the expression of IL-10 was observed in the head kidney of HI-supplemented groups 24-h post-challenge with *V. harveyi*, which may be associated with the decreased expression of IL-1β. The regulatory effect on the expression of IL-1β by IL-10 has been reported in Indian major carp, *Catla catla* (101). IL-10 plays a central role in suppressing inflammation through the inhibition of the production of pro-inflammatory cytokines (102), and expression is reported to increase in several fish species following LPS stimulation and bacterial and parasitic infections (103–105). Therefore, down-regulation of the expression level of IL-1β with concurrent up-regulation of IL-10 at 24-h challenge with bacteria indicates an active phase of anti-inflammatory effects and also suggests that HI-supplemented diets indirectly intervene in the response of the host to the pathogen by upregulating the expression level of IL-10.

## Conclusion

In summary, our research demonstrates that feeding juvenile barramundi with PBM and HI-larvae-supplemented diets for 6 weeks resulted in no significant impact on growth performances, biometry indices (except IFI), and also the integrity of liver, heart, and muscle. The IFI index declined in fish fed HI-supplemented diets as reflected by a decrease in the size of adipocyte cells. In response to 2 weeks of *V. harveyi* infection, survival rate augmentation in HI-supplemented PBM diets was further supported by a significant increase in serum immunity, skin-associated mucin production, and controlling inflammatory cytokines in response to 24-h post-challenge with bacteria. While the results are promising, further investigation is needed to decipher the role of chitin, antimicrobial peptides, and/or bioactive polysaccharides in HI larvae in influencing fish health.

## Data Availability Statement

The original contributions presented in the study are included in the article/supplementary material, further inquiries can be directed to the corresponding author/s.

## Ethics Statement

The animal study was reviewed and approved by Curtin University Animal Ethics Committee.

## Author Contributions

RF and JH conceptualized the experimental design and reviewed the manuscript. MC was involved in experimental design, conducting experiment, collecting samples, analyzing data, making table and figures, and writing the original draft. MS helped in formal analysis and proofreading the manuscript. All authors contributed to the article and approved the submitted version.

## Conflict of Interest

The authors declare that the research was conducted in the absence of any commercial or financial relationships that could be construed as a potential conflict of interest.
